# Genetics and Grain Filling Effects on Starch Properties in Wheat

**DOI:** 10.3390/foods15020357

**Published:** 2026-01-19

**Authors:** Yue Zhao, Yingkun Wang, Liwen Meng, Wenjie Li, Henan Wang, Qin Yan, Na Niu, Lingjian Ma

**Affiliations:** College of Agronomy, Northwest A&F University, Yangling 712000, China; zy1090644355@nwafu.edu.cn (Y.Z.); 15890951592@163.com (Y.W.); mengliwen2001@163.com (L.M.); 18435926328@163.com (W.L.); 18729255705@163.com (H.W.); 18893541961@163.com (Q.Y.); niuna@nwsuaf.edu.cn (N.N.)

**Keywords:** wheat, grain filling, mutant, starch, physicochemical properties, genetic effects

## Abstract

The physicochemical properties of wheat (*Triticum aestivum* L.) determine its application. This study aims to investigate the genetic patterns and heterosis performance of physicochemical properties of wheat starch, using the variety Xinong99 and two filling-deficient mutants, *dwarf wrinkle grain (dwg)* and *dwarf narrow grain (dng)*, obtained by EMS mutagenesis, as well as their backcross F1 generations, to systematically compare differences in starch structure, pasting characteristics, and thermal properties. The results showed that the mutants exhibited higher relative crystallinity, significantly reduced starch content and gelatinization temperature, while the gelatinization enthalpy and breakdown viscosity were significantly increased. Some F1 generation germplasm exhibited transgressive heterosis in viscosity characteristics; for example, the *dng* F1 had peak viscosity (4669 cP) and breakdown viscosity (2618 cP) higher than the parents, but its gelatinization enthalpy (2.29 J/g) was significantly lower than both parents. Furthermore, the *dwg* mutant starch granules had a more compact growth ring structure and showed higher resistance to digestion potential. This study systematically reveals the mechanisms of trait formation in mutants and hybrid offspring from the dimensions of starch structure–function–genetics, providing a theoretical basis and germplasm foundation for improving wheat starch quality through molecular design breeding.

## 1. Introduction

Wheat (*Triticum aestivum* L.) is one of the world’s major staple crops, ranking as the third most important grain crop in China’s agricultural production structure. Its grains are rich in various nutritional components such as starch, protein, and lipids, with starch being particularly prominent [1]. As the primary storage form of carbohydrates in grains, starch not only provides essential energy for the human body but also plays crucial roles in food processing and industrial applications [2,3]. In recent years, transgenic technology has been widely applied for the precise improvement of wheat starch quality. Through the regulation of key genes involved in starch synthesis, optimization of starch composition, granule structure, and functional properties has been achieved [4,5]. However, transgenic crops face stringent regulatory restrictions and public acceptance challenges during promotion and application, prompting researchers to shift towards non-transgenic breeding strategies. Approaches such as mutagenesis breeding combined with efficient screening techniques are being utilized to develop novel germplasm with superior starch traits, thereby avoiding transgenic controversies and expanding feasible pathways for wheat quality improvement [6,7,8,9]. Starch accounts for over 80% of the weight of the wheat endosperm, and its content, structure, and composition are key factors determining grain yield and quality [10]. The grain filling stage is the critical period for starch synthesis and accumulation. This process involves sucrose transport, carbohydrate metabolism, and multiple enzymatic reactions, ultimately leading to the formation and storage of starch [11,12,13,14]. Studies have indicated that mutations in key genes during the grain filling process can severely impair starch accumulation, consequently leading to reduced yield and quality. Examples include the *Grain Incomplete Filling 1 (GIF1)* gene in rice and the *Miniature 1 (Mn1)* gene in maize [15,16,17,18]. Abiotic stresses such as drought and high temperature can also inhibit starch synthase activity, affecting starch accumulation and composition [19]. Furthermore, significant differences exist among different wheat varieties in the expression of starch synthesis genes and starch structure, which consequently affect their physicochemical properties and end-use applications [20,21]. Filling deficiencies may also lead to an imbalance in starch type ratios and morphological changes in starch granules, affecting functional properties such as swelling power and gelatinization temperature [17]. Therefore, in-depth analysis of the regulatory mechanisms governing starch accumulation during the grain filling process is of great significance for the genetic improvement of wheat starch quality.

The physicochemical properties of starch, including pasting characteristics, thermal stability, and swelling power, are closely related to its fine structure and directly influence the processing quality and end-use of flour [22]. Research has shown that chain length distribution, crystalline type, and granule morphology of starch vary significantly among different varieties, leading to distinct functional properties [23]. For instance, through the analysis of 272 wheat varieties worldwide, researchers found significant correlations between grain hardness, total starch content, and starch digestibility [24]. Additionally, structural characteristics of starch granules, such as growth ring density and granule size, also influence their digestibility and processing performance [25,26,27]. In recent years, EMS mutagenesis has provided an effective means for creating wheat starch mutants. The screening and identification of mutants with high or low starch content and high resistant starch content, for example, have established a germplasm foundation for elucidating starch synthesis mechanisms [6,7]. However, systematic studies on starch structure and functional properties in grain filling-deficient mutants remain relatively scarce. In particular, the genetic inheritance patterns and heterosis performance of starch traits in their hybrid progeny are still unclear.

Based on this, this study selected two EMS-induced grain filling-deficient wheat mutants (*dwg* and *dng*) and their backcross F1 generations to systematically compare their differences with the parent in terms of starch structure, thermal properties, and pasting characteristics. The research aims to reveal the impact mechanisms of filling deficiency on starch quality formation, clarify its genetic inheritance patterns, and explore whether heterosis exists for starch traits in the hybrid progeny, thereby providing new germplasm and a theoretical basis for the non-transgenic genetic improvement of wheat starch quality.

## 2. Materials and Methods

### 2.1. Materials

**Figure 1:**
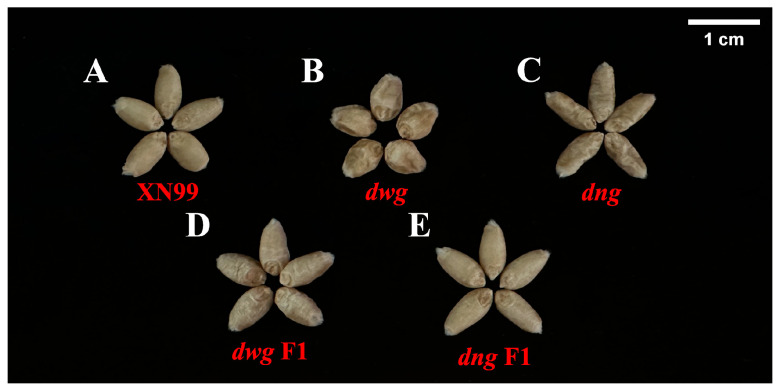
Grain morphology of different germplasms used in this study. (**A**) XN99, (**B**) *dwg*, (**C**) *dng*, (**D**) *dwg* F1 and (**E**) *dng* F1 represent Xinong99, the grain filling-deficient mutants *dwg* and *dng*, and their backcross F1 generations (*dwg* F1 and *dng* F1) with XN99, respectively.

In 2017, our research group first mutagenized Xinong99 (XN99) grains using 1.2% EMS with an 8 h treatment duration, obtaining 2377 M0 generation mutant germplasm. In 2019, phenotypic screening and identification were conducted on the M2 generation mutants, followed by four consecutive generations of self-pollination to stabilize the phenotypes. Ultimately, two stably inherited grain filling-deficient mutants, *dwarf wrinkle grain (dwg)* and *dwarf narrow grain (dng)*, were identified from the mutant library. *dwg* F1 and *dng* F1 are the F1 generations derived from crosses between *dwg* and XN99, and *dng* and XN99, respectively, and were used for analyzing their genetic effects, as shown in Figure 1. All germplasms were cultivated at the experimental station of Northwest A&F University (34°28′ N, 108°07′ E, approximately 530 m altitude) and used after harvest in 2023. After harvest, the samples were dried at room temperature (25 ± 5 °C) to ensure the grain moisture content was maintained at 10 ± 2%.

### 2.2. Measurement of Wheat Grain Traits

The grain phenotype and quality characteristics of the five germplasms were analyzed using an automatic seed testing and thousand-kernel weight analysis system (SC-G, Hangzhou Wanshen Testing Technology Co., Ltd., Hangzhou, China) and a near-infrared analyzer (DA7250, Perten, Stockholm, Sweden). The grain hardness index of 300 wheat kernels was determined using a Single Kernel Characterization System (SKCS 4100, Perten Instruments North America Inc., Springfield, IL, USA). According to the AACC55-31 method, varieties are typically classified into distinct categories based on hardness index criteria, including soft wheat (HI < 45), mixed/medium-hard wheat (45 ≤ HI ≤ 60), and hard wheat (HI > 60).

### 2.3. Preparation of Starch Samples

#### 2.3.1. Isolation of Wheat Starch

Total starch was extracted using a dough-washing method with modifications [28]. Wheat flour passed through a 100-mesh sieve was mixed with distilled water at a ratio of 10:7 to form a dough. The dough was rested at 25 °C for 15 min and then hand-washed to separate the starch. The suspension was passed through a 200-mesh sieve. The filtrate was centrifuged at 2500× *g* for 10 min. The supernatant was discarded, and the top layer was scraped off. The precipitate was washed and centrifuged three times each with 2% sodium dodecyl sulfate (SDS) and 2 M sodium chloride, followed by washing with distilled water until foam-free. The final white precipitate was dried at 35 °C for 12 h, ground into powder, passed through a 200-mesh sieve, and stored at 4 °C for later use.

#### 2.3.2. Separation of A-Type and B-Type Starch

A- and B-type starch granules were separated as described by Zeng et al. (2011) [27] with slight modifications. One gram of wheat starch was weighed and mixed with an equal volume of distilled water (1:4, *w*/*v*). The mixture was stirred to disperse the starch granules, allowed to stand for 5 min, and then the upper suspension was centrifuged at 1308× *g* for 10 min and dried to obtain wheat B-starch. The bottom precipitate was dried to obtain wheat A-starch.

### 2.4. Morphological Characteristics

#### 2.4.1. Polarized Light Microscopy (PLM)

A small amount of glycerol and water (1:1, *v*/*v*) was dropped onto the starch sample on a glass slide. The sample was observed under a polarized light microscope (NIKON Eclipse ci, Nikon Precision (Shanghai) Co., Ltd., Shanghai, China) at 200× magnification [29,30,31].

#### 2.4.2. Scanning Electron Microscopy (SEM) Analysis

A small number of dry starch granules was mounted on a specimen stub using black double-sided adhesive tape. After gold coating under vacuum, the morphological structure of the starch granules was observed at a suitable magnification and analyzed at 15 keV using a scanning electron microscope (SEM, TM4000Plus, Hitachi, Japan), as described by Li et al. (2014) [32].

### 2.5. Analysis of Starch Shell Structure by Enzymatic Hydrolysis-SEM

A starch sample (1 g, dry basis) was mixed with 10 mL of α-amylase solution (80 U/mL) in a 100 mL Erlenmeyer flask. The sample mixture was incubated in a water bath at 25 °C with stirring at 150 rpm for 12 h. Then, the sample was centrifuged at 3000× *g* for 10 min [33]. The starch shell structure was analyzed using SEM according to method 2.4.2. Analytical grade α-amylase (EC 3.2.1.1, 4000 U/g) was purchased from Beijing Solarbio Science & Technology Co., Ltd., Beijing, China.

### 2.6. Determination of Starch Granule Size Distribution

The granule size distribution of the starch samples was determined using an S3500 laser diffraction particle size analyzer (Microtrac Co., Ltd., Montgomeryville, PA, USA) with distilled water as the background medium, repeated three times. The instrument parameters were set as follows: refractive index of water, 1.330; refractive index of starch, 1.530; starch absorption index, 0.1 [34].

### 2.7. X-Ray Diffraction (XRD) Analysis

The diffraction patterns of the starch samples were measured using an X-ray diffractometer (D8 ADVANCE A25, Bruker-AXS, Karlsruhe, Germany). The instrument parameters were as follows: radiation source, Cu (voltage 40 kV, current 40 mA); diffraction scan range from 5° to 50° (2θ) with a step size of 0.02°; scan speed of 6°/min. The relative crystallinity was calculated as the percentage of the diffraction peak area to the total area using the software Jade 6.2 [30,35].

### 2.8. Determination of Amylose and Total Starch Content

The amylose content of the samples was determined using a Futura3 fully automatic amylose analyzer (AMS Co., Ltd., Rome, Italy). Total starch content was determined using an assay kit (Fankew, Shanghai Kexing Trading Co., Ltd., Shanghai, China). Amylopectin content = Total starch content − Amylose content.

### 2.9. Determination of Solubility and Swelling Power

The method was modified from Wang et al. (2020) [36]. Starch sample (m0) was mixed with water (2%, *w*/*v*) in a 2 mL centrifuge tube (m1) and heated in a water bath at 95 °C for 30 min. The sample was cooled to room temperature, centrifuged at 8000× *g* for 10 min, and the remaining gel in the tube was weighed (m2). The sediment was dried at 60 °C to constant weight (m3). Swelling power and solubility were calculated as follows:

Swelling power (g/g) = (m2 − m1)/(m3 − m1)

Solubility (%) = 100 × (m0 + m1 − m3)/m0

### 2.10. Light Transmittance

The light transmittance (LT) of starch pastes was determined with some modifications according to the method described by Gao et al. (2022) [37]. Briefly, a 1% starch suspension was boiled in a 100 °C water bath for 30 min with constant stirring. Thereafter, the starch paste was cooled to room temperature, and the LT value was measured at 620 nm using a UV spectrophotometer (Blue Star B, Lab Tech Ltd., Beijing, China) [36].

### 2.11. Pasting Properties Analysis

The pasting properties of the starch were analyzed using a Rapid Visco Analyzer (RVA 4500, Perten, Stockholm, Sweden). Starch sample (3 g, dry weight) was mixed with 25 g of distilled water. The mixture was heated to 50 °C and held for 1 min; then heated to 95 °C uniformly over 3.75 min and held for 2 min; subsequently cooled to 50 °C at a rate of 12 °C/min and held for 1 min [38].

### 2.12. Determination of Thermal Properties by Differential Scanning Calorimeter (DSC)

Starch sample (3 mg, dry basis) was mixed with 9 μL of distilled water. The analysis was performed with a nitrogen flow rate of 50 mL/min, by scanning from 20 to 120 °C at a heating rate of 10 °C/min. A differential scanning calorimeter (Q2000, TA Instruments, Newcastle, DE, USA) was used [30,39,40].

### 2.13. Statistical Analysis

One-way analysis of variance (ANOVA) followed by Duncan’s test was performed using SPSS software (SPSS 27.0.1.0, SPSS Inc., Chicago, IL, USA). The significance threshold was set at *p* < 0.05. All tests were performed with at least three replicates.

## 3. Results and Discussion

### 3.1. Characterization of Wheat Grain Traits

#### 3.1.1. Seed Testing Data of Wheat

**Figure 2:**
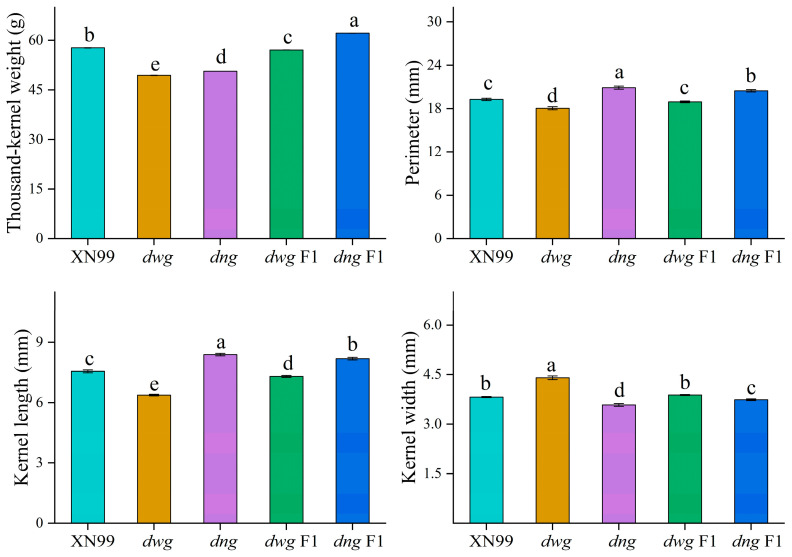
Grain characterization properties. Note: Data are presented as mean ± SD. Different lowercase letters within the same column indicate significant differences (*p* < 0.05, *n* = 3). The meaning of the abbreviation is as stated above.

As shown in Figure 2, the thousand-kernel weights (TKW) of the mutants *dwg* and *dng* were both around 50 g, while the TKW of XN99 was significantly higher than both mutants (*p* < 0.05). In terms of kernel shape, *dwg* exhibited obvious phenotypic changes. Its kernel length-to-width ratio was significantly lower than that of the wild type (*p* < 0.05), with a reduction of 25.76%, indicating a change from the typical oval shape to a nearly round shape (Figure 1, Table S1). In contrast, the kernel length of *dng* increased by 11.26%, but its kernel width decreased by 6.02%. The kernels of both mutants exhibited a shrunken phenotype with poor plumpness, which is consistent with their significantly reduced thousand-kernel weights. In fact, kernel weight is influenced not only by kernel size but also by the extent of grain filling [14,17]. Regarding genetic effects, the F1 generation derived from crossing *dwg* and *dng* exhibited epistatic effects on multiple traits. It is particularly noteworthy that the TKW of the *dng* F1 generation was significantly higher than that of the parent XN99 (*p* < 0.05), with an increase of 7.6%. This indicates that favorable alleles present in the mutant can significantly enhance the key yield trait of kernel weight in a heterozygous background. This result suggests that crosses between parents with large phenotypic differences may be more likely to yield heterosis, thereby providing an effective strategy for crop genetic improvement. As reported by Wang et al. (2008) [18] for the *GIF1* gene in rice, introducing this gene through crossing can effectively increase grain yield, and the yield performance of the progeny tends to favor the parent with superior genetic indicators [41].

#### 3.1.2. Wheat Hardness Index

Hard and soft wheat are classified based on the hardness index. Wheat with a hardness index ≥ 60 is classified as hard, while wheat with a hardness index ≤ 45 is classified as soft [42]. As shown in Table 1, the hardness indices of the five germplasms were approximately 50, and they were classified as medium-hard wheat. Regarding hardness distribution, their hardness profiles were relatively scattered. Compared to the wild-type XN99, *dng* showed a significantly reduced hardness index (*p* < 0.05) and was classified as class 03; *dwg* also exhibited a similar decreasing trend. The hardness indices of the hybrid F1 generations were similar to their corresponding mutant parents and did not exhibit significant heterosis. The above results indicate that changes in kernel morphology have a limited effect on kernel hardness, and the hardness trait did not show a dominant inheritance trend in the hybrid generations. This suggests that this trait is likely regulated by multiple factors, such as starch and protein composition and their interactions [42,43].

#### 3.1.3. Near-Infrared Quality Characteristics

Protein and starch are the main nutritional components in wheat grains, and their content and composition significantly influence the processing and nutritional quality of wheat [1]. The protein content in wheat grains generally ranges from 8% to 14% [2,10,17]. As shown in Table 2, the crude protein content (dry basis) of XN99 was approximately 15%. In comparison, the crude protein content of *dwg* and *dng* was significantly increased (*p* < 0.05), exceeding the common range for ordinary wheat. Concurrently, their starch content decreased accordingly, indicating a protein-starch trade-off effect in grain nutritional composition. The impediment of starch synthesis in the grains likely diverted resources towards protein production, ultimately leading to a significant increase in protein content [11]. The high-protein phenotype was accompanied by a significant improvement in gluten quality: *dng* had the highest wet gluten content, a sedimentation value significantly higher than other germplasm (*p* < 0.05), and significantly enhanced extensibility (*p* < 0.05). On the other hand, *dwg* and *dng* exhibited lower water absorption and a decreasing trend in test weight. Akel et al. (2019) [41] reported that heterosis for wheat grain yield can reach up to 10%, while heterosis for quality traits is lower or even negative, which is consistent with the results of this experiment. Among the hybrid F1 generations, *dwg* F1 showed higher values than the wild type for crude protein, gluten index, and sedimentation value, but still lower than its mutant parent; *dng* F1 did not exhibit significant heterosis for quality traits. This indicates that the genetic behavior of protein and related quality traits is complex and likely regulated by non-additive effects.

### 3.2. Wheat Starch Granule Size Distribution

**Figure 3:**
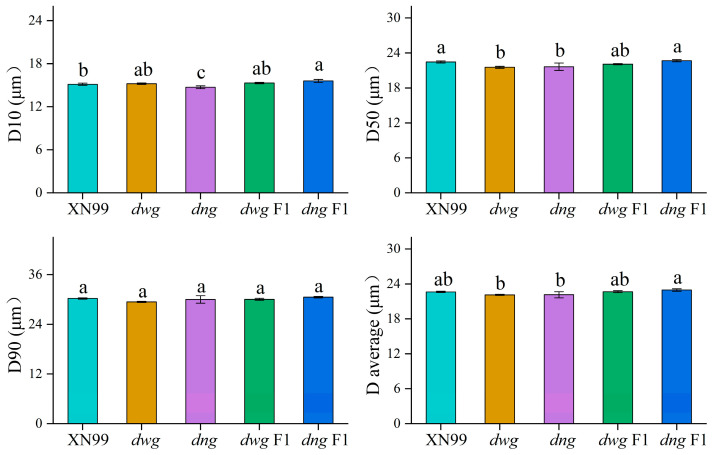
Size Distribution of Starch Granules. D10, the maximum particle diameter representing 10% of the size distribution; D50, the median diameter representing 50% of the size distribution; D90, the maximum particle diameter representing 90% of the size distribution; D[4,3], volume mean diameter. Note: Data are presented as mean ± SD. Different lowercase letters within the same column indicate significant differences (*p* < 0.05, *n* = 3). The meaning of the abbreviation is as stated above.

Granule size distribution influences the physicochemical properties of starch, which is reflected in its applications in both food and non-food industries [33]. The determined starch granule size distribution results for the five germplasms are shown in Figure 3. The starch granules of common wheat XN99 exhibited a unimodal distribution, and no significant differences (*p* > 0.05) were observed in its volume mean diameter D[4,3] compared to the other four germplasms. Both mutants exhibited smaller particle sizes in the D10, D50, and D90 parameters, but their sphericity was consistent with the common wheat germplasm, showing no significant change. In the hybrid F1 generations, the D[4,3] values of *dwg* F1 and *dng* F1 were 22.68 μm and 22.97 μm, respectively, both significantly higher than their corresponding mutant parents (*p* < 0.05), and not significantly different from the wild-type XN99. Furthermore, *dng* F1 was significantly higher than *dng* in the D50 parameter (*p* < 0.05), indicating a certain recovery effect of the hybrid generation on the granule size trait. Changes in the size distribution of starch granules not only affect physicochemical properties, but are also closely related to wheat processing quality [44]. Studies have found that particle size significantly influences the stability of dispersion systems, with smaller particles conferring higher stability [34].

### 3.3. Starch Granule Morphological Properties

#### 3.3.1. Polarized Light Microscopy (PLM) Analysis

**Figure 4:**
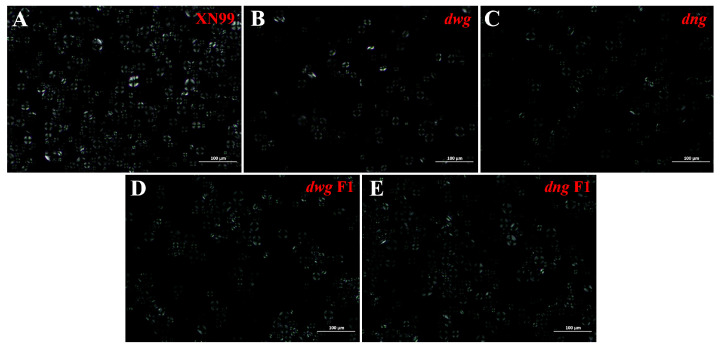
Polarized light observation of starch granules. Note: The meaning of the abbreviation is as stated above. (**A**) XN99, (**B**) *dwg*, (**C**) *dng*, (**D**) *dwg* F1 and (**E**) *dng* F1 represent Xinong99, the grain filling-deficient mutants *dwg* and *dng*, and their backcross F1 generations (*dwg* F1 and *dng* F1) with XN99, respectively.

Polarized light microscopy (PLM) images of starch granules from each sample are shown in Figure 4. Due to differences in density and refractive index between the crystalline and amorphous regions within starch granules, anisotropy occurs when polarized light passes through, resulting in the typical “Maltese cross” birefringence pattern. The intensity of this pattern reflects the integrity and degree of order of the granular crystalline structure [29,30,31,43]. As shown in Figure 4, the starch granules of common wheat cultivar XN99 displayed clear and bright birefringence patterns, indicating an intact crystalline structure with a high degree of order. In contrast, the birefringence intensity of the *dwg* and *dng* mutants was significantly weakened, indicating a reduction in the structural order or crystalline integrity of their crystal lattice. The birefringence intensity of the hybrid F1 generation starch granules was intermediate between the corresponding mutants and the common wheat parents, showing a partial recovery trend. The birefringence of XN99 granules was greater and sharper, likely due to a higher hierarchy of crystalline organization [29].

#### 3.3.2. Morphological Analysis of Starch Granules by SEM

**Figure 5:**
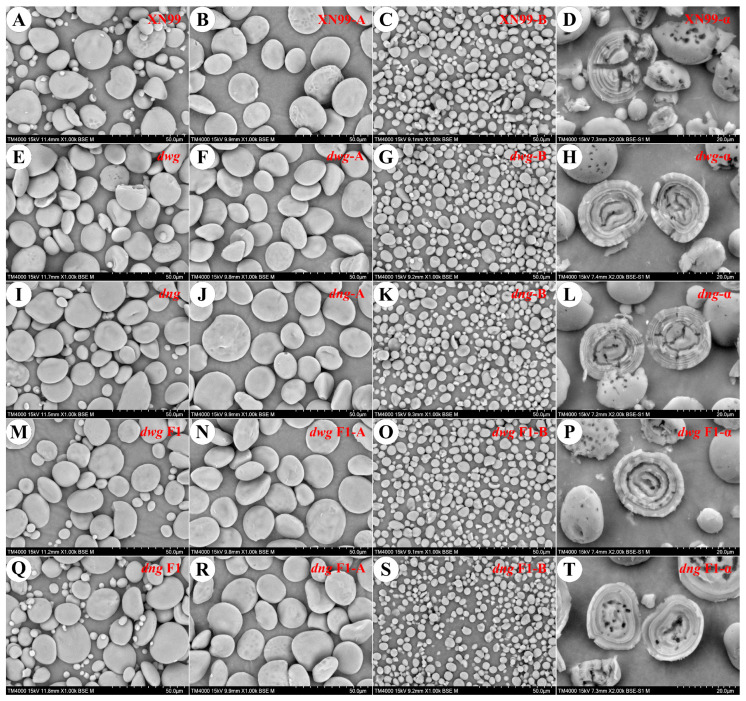
Scanning electron microscopy of total starch, A-type starch, B-type starch, and α-amylase-hydrolyzed starch shells. Note: XN99 (**A**–**D**), *dwg* (**E**–**H**), and *dng* (**I**–**L**) represent Xinong99 and the grain filling-deficient mutants *dwg* and *dng*, respectively. *dwg* F1 (**M**–**P**) and *dng* F1 (**Q**–**T**) are the backcross F1 generations of *dwg* and *dng* with XN99, respectively.

The morphology of wheat starch granules is an important factor influencing their functional properties. Typically, based on granule size, wheat starch can be divided into two types: A-type granules, which are lenticular in shape with a diameter between 20 and 45 μm, accounting for about 70–80% of the total starch mass; and B-type granules, which are mostly spherical with a diameter less than 10 μm, usually accounting for less than 10% [45]. Observations by scanning electron microscopy (SEM) (Figure 5) showed that the A-type starch granules of common wheat cultivar XN99 possessed obvious surface indentations. In contrast, the A-type granules of the *dwg* and *dng* mutants had smoother surfaces with reduced indentation; their proportion of B-type granules was lower, with greater size variation and poorer uniformity. In the hybrid F1 generations, both *dwg* F1 and *dng* F1 partially recovered the indentation features on the surfaces of the A-type granules. It is noteworthy that *dng* F1 had a higher proportion of B-type granules and a more uniform size distribution, with its overall performance being superior to that of XN99 and *dwg* F1. This result is consistent with the findings of Sun et al. (2021) [33]. The differences in the microscopic surface morphology of wheat A- and B-starch granules may be attributed to genetic factors regulating starch biosynthesis, the ratio of amylose to amylopectin, molecular composition, and other factors.

#### 3.3.3. Structural Characteristics of Starch Shell

Starch granules possess a multi-level hierarchical structure, comprising the intact granule as a whole, and periodically alternating crystalline and amorphous growth rings. Acid or enzymatic hydrolysis treatments combined with scanning electron microscopy (SEM) imaging can further reveal the lamellar structure and hydrolysis patterns of starch [46,47,48]. Among these, semi-crystalline growth rings (with a repeat period of ~9–10 nm) and amorphous growth rings (~60–80 nm wide) together form a concentric shell-like annual ring structure [26,33]. After α-amylase hydrolysis treatment, wheat starch granules showed significant fragmentation. The degree of damage was higher in A-type than in B-type starch, exposing a clear shell-like structure (Figure 5), indicating that enzymatic hydrolysis preferentially occurs in the amorphous regions. The growth ring structures of XN99 exhibited relatively large amorphous regions, with a pronounced alternation between amorphous and semi-crystalline structures. The semi-crystalline regions of the *dwg* and *dng* mutants were denser than those of XN99; furthermore, the growth ring density of their hybrid F1 generations (*dwg* F1 and *dng* F1) increased further, being significantly higher than that of the parental germplasm. Highly dense growth ring structures and smaller amorphous regions typically indicate higher structural order and crystallinity of the starch [33,48], which may be related to its physicochemical properties (such as pasting characteristics, swelling power, and digestibility).

### 3.4. Crystalline Structure Analysis

**Figure 6:**
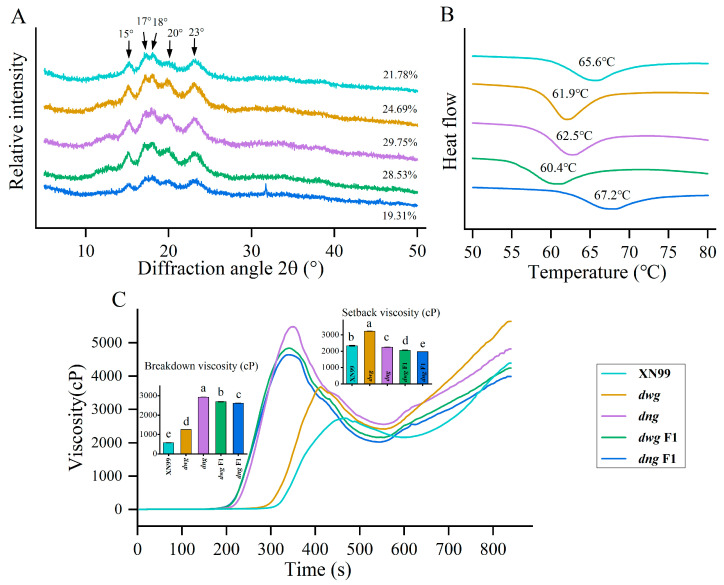
Physicochemical properties of the five germplasms: (**A**) XRD patterns, (**B**) DSC thermograms, (**C**) RVA pasting profiles. Note: Different letters indicate significant differences at *p* < 0.05. The meaning of the abbreviation is as stated above.

Based on X-ray diffraction patterns, starch crystalline structures can be classified into four types: A, B, C, and V. All five wheat germplasms exhibited a typical A-type crystalline structure, with characteristic diffraction peaks at 2θ = 15°, 17°, 18°, and 23°, where the peaks at 17° and 18° form a connected doublet (Figure 6A). Additionally, a weak diffraction peak was detected at 2θ = 20° in all samples, which is characteristic of an amylose-lipid complex [29]. The relative crystallinity of the various samples ranged from 17.31% to 29.75% (Figure 6A). The *dng* mutant showed higher diffraction peak intensity, and its relative crystallinity was significantly higher than that of the other genotypes (*p* < 0.05). Conversely, *dng* F1 exhibited the weakest diffraction intensity and the lowest crystallinity. The relative crystallinity of *dwg* F1 was significantly higher than that of both its parents (*p* < 0.05), indicating significant epistatic genetic effects for this trait [49]. Starch crystallinity is closely related to its granule structure and thermal properties [25]. The results of this study suggest that higher crystallinity may reflect a more ordered molecular arrangement and a more stable structure, which is consistent with the reported influence of crystallinity on pasting properties and digestive resistance by Liu et al. (2023) [16].

### 3.5. Total Starch and Amylose Content

In mature wheat grains, starch accounts for about 65% and is the main component. Its content and composition directly affect the physicochemical properties of the starch [28,29,44]. As shown in Table 3, except for *dwg* and *dng*, whose total starch content was significantly reduced due to grain shrinkage (*p* < 0.05), the total starch content of the other germplasm ranged between 66.27% and 69.02%. Regarding starch composition, *dwg* had the highest amylose content (30.63 ± 0.07%), and its amylose-to-amylopectin ratio was 0.442, significantly higher than that of other genotypes (*p* < 0.05). The amylose-to-amylopectin ratio of *dwg* F1 was significantly lower than that of both parents (*p* < 0.05), exhibiting a certain non-additive genetic effect; *dng* F1 showed no significant difference from its parents. In wheat starch, amylose accounts for about 15–25%, and amylopectin for about 75–85%. The amylose-to-amylopectin ratio is an important indicator reflecting starch digestibility [50]. Amylose is important for the formation of starch gels, while amylopectin influences the retrogradation, gelatinization, acid hydrolysis, enzymatic hydrolysis, and rheological properties of starch granules [2,10].

### 3.6. Light Transmittance, Swelling Power, and Solubility

The transparency of starch paste reflects the strength of its water-binding capacity. The extent of molecular rearrangement and reassociation after gelatinization is an important factor affecting paste transparency, which is quantified by light transmittance. A higher light transmittance indicates better starch paste transparency [16,36]. The light transmittance of starch paste can reflect the degree of interaction between starch molecules and water and their rearrangement after gelatinization. As can be seen from Table 3, the light transmittance of wheat starch from different genotypes showed significant differences (*p* < 0.05). Light transmittance showed a negative correlation trend with crude protein content (Table 2): the mutants *dwg* and *dng* had the highest crude protein content but the lowest light transmittance; conversely, *dng* F1 had a light transmittance of 98.8%, significantly higher than all other germplasm (*p* < 0.05), exhibiting obvious transgressive heterosis. This trend in results is similar to the research reported by Gao et al. (2022) [37]. The swelling power and solubility of starch are related to the content of components such as amylose, amylopectin, protein, and lipids, as well as granule size in wheat starch [30,36]. There were also significant differences in swelling power and solubility among the germplasm (*p* < 0.05). The two mutants *dwg* and *dng* exhibited the highest and lowest swelling power, respectively. The swelling power and solubility of their F1 generations were mostly intermediate between the parental values and did not show significant transgressive heterosis, indicating that these traits are likely governed by additive gene effects.

### 3.7. Pasting Properties by RVA

The pasting properties of starch are its important functional characteristics and are closely related to granule structure and molecular composition [25,30,44]. Significant differences (*p* < 0.05) were observed between the common wheat cultivar XN99 and the mutants in parameters such as peak viscosity, trough viscosity, and final viscosity (Table S2). As shown in Figure 6C, compared to the wild type, *dwg*, *dng*, and their F1 generations had significantly higher peak viscosity and trough viscosity (*p* < 0.05). The breakdown viscosity (BV) differed significantly among germplasm, ranging from 577 to 2925 cP, with XN99 being the lowest and *dng* the highest (Figure 6C). The setback viscosity (SV) ranged from 1972 to 3233 cP, with *dng* F1 being the lowest and *dwg* the highest (Figure 6C). Regarding pasting temperature, *dng*, *dwg* F1, and *dng* F1 were all significantly lower than other genotypes (*p* < 0.05), which might be related to higher crystallinity promoting water penetration and the gelatinization process, consistent with the X-ray diffraction analysis results. The complexity of starch gelatinization implies that Tp is influenced by multiple factors, not limited to amylose content. The fine structure of amylopectin (chain length distribution) and starch granule architecture (e.g., crystallinity) may play significant roles in determining Tp, a pattern also confirmed by the research of Huang et al. (2022) [2]. We propose that, in the specific mutant lines of this study, alterations in these other structural parameters may have masked or counterbalanced the effect of amylose content variation, thereby leading to the observed lack of correlation. *dwg* F1 exhibited transgressive heterosis in both peak viscosity and setback value. Its breakdown viscosity was significantly higher than both parents, while its setback value was significantly lower (*p* < 0.05). This trait performance might be related to its observed dense growth ring structure. The hybrid F1 generations exhibited different genetic patterns: the pasting properties of *dng* F1 showed mid-parent inheritance, while *dwg* F1 showed transgressive heterosis in multiple parameters, indicating that pasting traits are regulated by different genetic effects [8,49].

### 3.8. Thermal Properties of Starch

The gelatinization thermal properties parameters of starch from the five germplasms, including onset temperature (To), peak temperature (Tp), conclusion temperature (Tc), and enthalpy of gelatinization (ΔH), were determined using a differential scanning calorimeter (DSC). The DSC curves of all samples showed smooth profiles (Figure 6B), and the thermodynamic parameters are shown in Table S3. The To, Tp, and Tc of the *dwg* and *dng* mutants were all significantly higher than those of the wild-type XN99 (*p* < 0.05). Among the hybrid F1 generations, *dwg* F1 had the lowest Tp (60.39 °C), while *dng* F1 had the highest Tp (67.16 °C). The enthalpy of gelatinization (ΔH) ranged from 2.41 to 2.79 J/g. The ΔH values of *dwg* and *dng* were significantly higher than those of XN99 (*p* < 0.05), but the ΔH of their corresponding F1 generations were all lower than those of both parents, exhibiting significant negative transgressive heterosis. The above results indicate that the thermal properties of starch differ significantly among genotypes, and the hybrid F1 generations did not show an advantage in the gelatinization enthalpy trait, suggesting that this trait may be regulated by non-additive genetic effects [51]. EMS mutagenesis typically generates thousands of SNP point mutations across the genome. Backcrossing the mutants to the mutagenized parent, XN99, is an effective genetic purification strategy that simplifies the mutant genetic background, thereby enhancing the accuracy of genetic analysis and breeding efficiency. If the observed starch trait variations were due to random background mutations, the probability would be extremely low for two independent mutants to simultaneously exhibit similar and significant alterations in starch properties (e.g., higher breakdown viscosity, higher gelatinization enthalpy, etc.) and for their backcross F1 progeny to display specific inheritance patterns. A more plausible explanation is that the two mutants carry independent, yet functionally similar, mutations in key pathways affecting grain filling and starch synthesis.

## 4. Conclusions

Through comprehensive analysis of wheat grain filling-deficient mutants and their hybrid F1 generations, this study elucidated the mechanisms and genetic patterns underlying starch trait formation. The main findings include, at the microstructural level, the mutant starches exhibited higher relative crystallinity than the mutagenized parent; in terms of pasting properties, the mutants demonstrated decreased gelatinization temperature and increased breakdown viscosity, whereas the F1 generations exhibited transgressive heterosis in viscosity and retrogradation properties; and regarding genetic inheritance, different functional properties of starch displayed diverse genetic patterns in the hybrid progeny. However, it should be noted that F1 seeds exhibit genetic segregation, and the results partially reflect the average phenotypic performance. This study focuses on the average starch properties of the F1 hybrid seed bulk and their potential application value (e.g., for direct processing), rather than their characteristics as stable breeding lines. However, fixing these advantages would require subsequent selection in segregating generations and genotype identification. This study provides important theoretical support for the genetic improvement of wheat starch quality, and offers crucial F1 generation evidence and insights into parental selection strategies for enhancing wheat starch quality through heterosis utilization.

## Figures and Tables

**Table 1:** Grain Hardness Characteristics.

Samples	Hardness Index	Moisture (%)	Soft	Semi-Soft	Semi-Hard	Hard	Classing Index
XN99	54.39 ± 13.01a	10.88 ± 0.25b	6%	18%	37%	39%	01
*dwg*	53.72 ± 14.83a	10.09 ± 0.40c	7%	23%	35%	35%	01
*dng*	49.01 ± 15.80b	9.16 ± 0.67d	15%	30%	29%	26%	03
*dwg* F1	54.31 ± 12.47a	11.02 ± 0.33b	5%	20%	41%	34%	01
*dng* F1	50.95 ± 12.73b	11.39 ± 0.30a	8%	28%	37%	27%	01

Note: Data are presented as mean ± SD. Different lowercase letters within the same column indicate significant differences (*p* < 0.05, *n* = 3). The meaning of the abbreviation is as stated above.

**Table 2:** Grain Quality Characteristics Determined by Near-Infrared Spectroscopy.

Samples	Crude Protein (%)	Gluten Index (%)	Water Absorption (%)	Sedimentation Value (g/L)	Stability Time (min)	Development Time (min)	Extensibility	Test Weight (g/L)
XN99	14.61 ± 0.05e	30.83 ± 0.15e	60.53 ± 0.15b	43.50 ± 0.20e	31.40 ± 0.10d	4.80 ± 0.10e	150.93 ± 2.25d	786.84 ± 0.89a
*dwg*	19.20 ± 0.03b	36.40 ± 0.16b	56.67 ± 0.06d	54.30 ± 0.17b	29.27 ± 0.06e	5.73 ± 0.06c	172.87 ± 0.31b	739.72 ± 1.46c
*dng*	22.48 ± 0.03a	44.59 ± 0.06a	56.37 ± 0.06e	69.90 ± 0.17a	51.97 ± 0.15a	9.73 ± 0.06a	219.33 ± 0.75a	785.93 ± 1.64a
*dwg* F1	17.71 ± 0.16c	35.81 ± 0.19c	59.47 ± 0.15c	51.73 ± 0.38c	35.50 ± 0.30b	6.57 ± 0.12b	172.80 ± 2.00b	751.62 ± 1.83b
*dng* F1	15.59 ± 0.04d	32.90 ± 0.09d	61.23 ± 0.15a	46.67 ± 0.12d	31.90 ± 0.17c	5.40 ± 0.10d	158.93 ± 1.21c	783.96 ± 0.77a

Note: Data are presented as mean ± SD. Different lowercase letters within the same column indicate significant differences (*p* < 0.05, *n* = 3). The meaning of the abbreviation is as stated above.

**Table 3:** Total starch content and composition, Swelling power, Solubility, Light transmittance.

Samples	Total Starch (%)	AM(%)	AP(%)	AM/AP	Swelling Power (g/g)	Solubility (%)	Light Transmittance (%)
XN99	69.02 ± 0.19a	29.17 ± 0.11b	70.83 ± 0.11b	0.412 ± 0.002b	17.12 ± 0.15c	17.41 ± 0.22b	68.7 ± 0.20b
*dwg*	64.65 ± 0.12d	30.63 ± 0.07a	69.37 ± 0.07c	0.442 ± 0.002a	16.15 ± 0.35d	15.91 ± 0.26c	30.9 ± 0.10d
*dng*	61.91 ± 0.04e	29.12 ± 0.14b	70.88 ± 0.14b	0.411 ± 0.003b	20.26 ± 0.20a	20.08 ± 0.33a	31.2 ± 2.60d
*dwg* F1	66.27 ± 0.15c	27.97 ± 0.14c	72.03 ± 0.14a	0.388 ± 0.003c	19.54 ± 0.20b	16.28 ± 0.44c	34.1 ± 1.20c
*dng* F1	67.88 ± 0.17b	29.11 ± 0.07b	70.89 ± 0.07b	0.410 ± 0.002b	15.36 ± 0.23e	15.86 ± 0.56c	98.8 ± 0.30a

Note: Data are presented as mean ± SD. Different lowercase letters within the same column indicate significant differences (*p* < 0.05, *n* = 3). The meaning of the abbreviation is as stated above.

## Data Availability

The original contributions presented in the study are included in the article/Supplementary Materials; further inquiries can be directed to the corresponding author.

## Supplementary Materials

The following supporting information can be downloaded at: https://www.mdpi.com/article/10.3390/foods15020357/s1, Table S1: Grain and starch properties of the five wheat germplasms; Table S2: Pasting properties of starch from the five wheat germplasms; Table S3: Thermal properties of starch from the five wheat germplasms.
